# Supplementary material for: Unveiling the critical role of androgen receptor signaling in avian sexual development
Source: Nat Commun. 2024 Oct 17;15:8970. doi: 10.1038/s41467-024-52989-w (PMC11487053; doi:10.1038/s41467-024-52989-w)
Supplement: Supplementary file 1 — Supplementary Information [file 41467_2024_52989_MOESM1_ESM.pdf]

# Supplementary Materials for

**Unveiling the critical role of androgen signaling in avian sexual development.**

Kamila Lengyel[1,2#], Mekhla Rudra[1#], Tom V.L. Berghof[2], Albertine Leitão[1], Carolina Frankl-Vilches[1], Falk Dittrich[1], Denise Duda[2], Romina Klinger[2], Sabrina Schleibinger[1], Hicham Sid[2], Lisa Trost[1], Hanna Vikkula[2], Benjamin Schusser*[2], Manfred Gahr*[1]

1: Department of Behavioural Neurobiology, Max Planck Institute for Biological Intelligence, Seewiesen, Germany. 2: Reproductive Biotechnology, TUM School of Life Sciences Weihenstephan, Technical University of Munich, Freising, Germany.

#: authors contributed equally

*: these authors jointly supervised this work

Correspondence to: manfred.gahr@bi.mpg.de

**This file includes:**

Figures S1 to S7

Table S1 to S7

## Supplementary figures

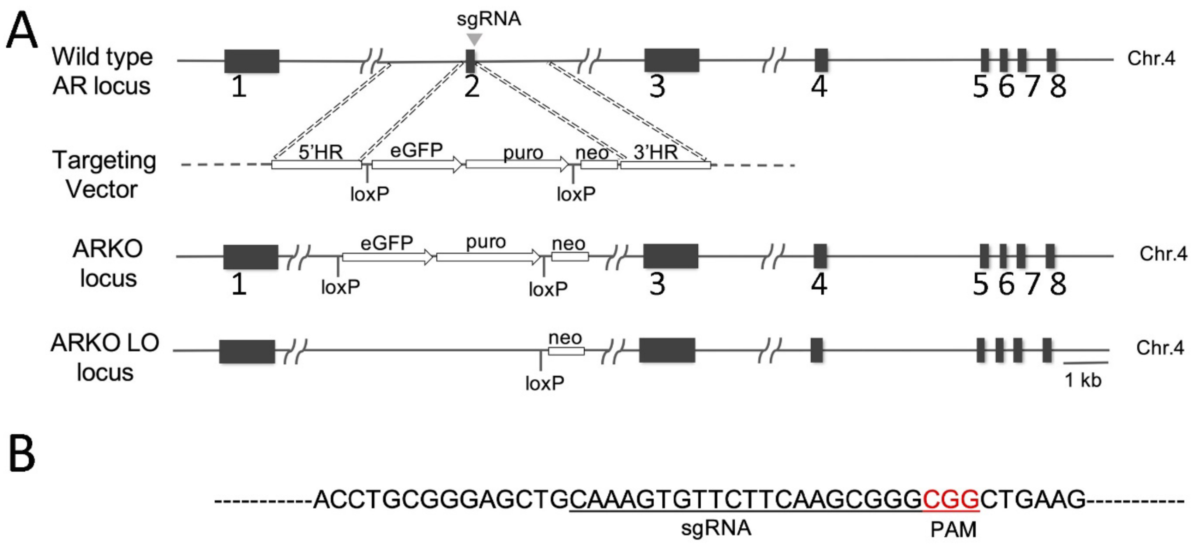


**Fig. S1. A.** The gene targeting strategy involved the deletion of exon 2, which codes for the DNA binding domain of the androgen receptor (AR). The wild-type AR locus consists of eight exons located on chicken chromosome 4 (Chr. 4). To generate AR knockout ($AR^{-/-}$) clonal primordial germ cells, a specific single guide RNA (sgRNA) was designed. A targeting vector was constructed with 5' and 3' homologous arms, along with a selectable marker cassette. The CRISPR/Cas9 system and homologous directed repair were used in this process. **B.** Sequence of the specific guide RNA (sgRNA) and the PAM site. sgRNA was designed with the online platform Benchling.

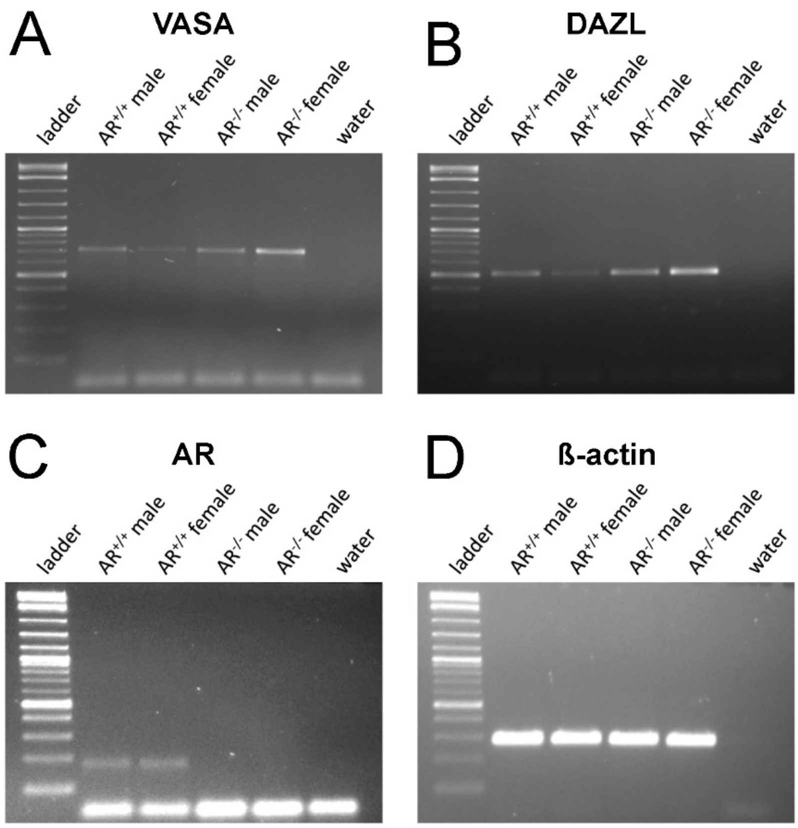


**Fig. S2: Analysis of the expression of VAZA, DAZL, AR, and β-actin mRNA in the gonad of male and female 18-day embryos, detected by PCR.** Both, wild-type ($AR^{+/+}$) and homozygous knockout ($AR^{-/-}$) male and female gonads showed expression of the germ cell markers VASA (**A**) and DAZL (**B**). These results indicate the presence of primordial germ cells in the testis and ovary, unaffected by the AR mutation. Given that the AR mutation was made by looping out exon 2, the AR mRNA was expected being absent in $AR^{-/-}$ samples with the used primer pair for exons 2 and 3 (**C**). Water served a negative control and ß-actin (**D**) as a positive control.

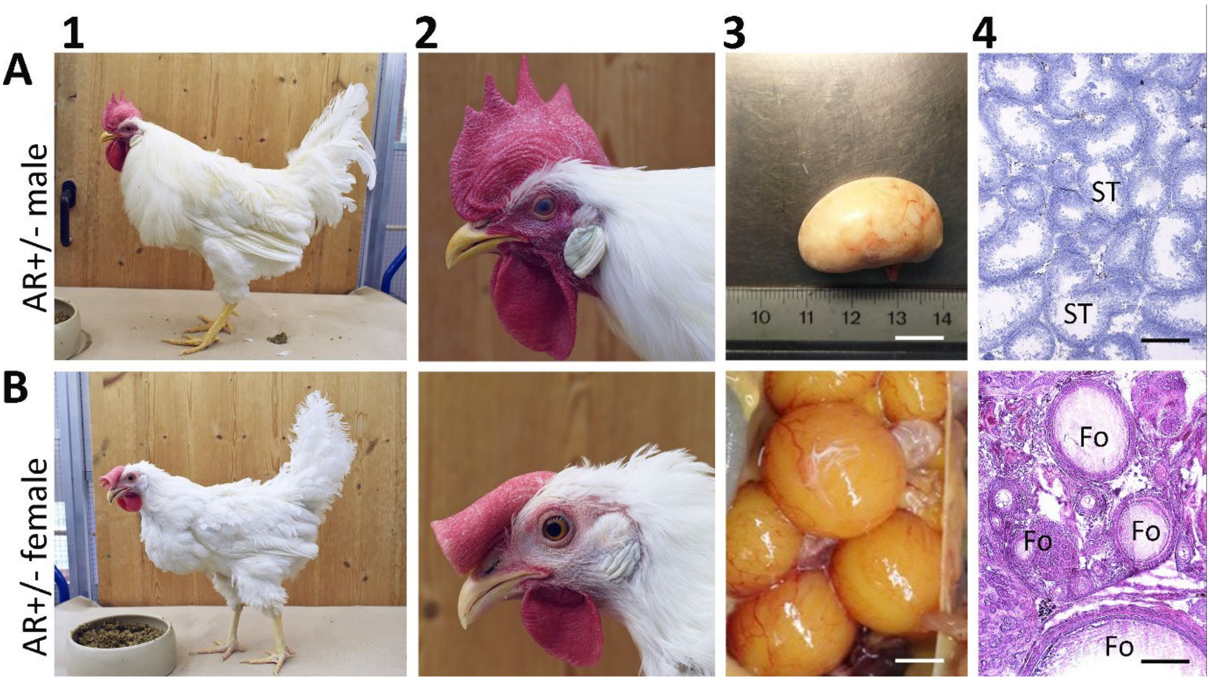


**Fig. S3. Heterogenous ($AR^{+/-}$) male and female sexual phenotypes are similar to that of wild-types.** $AR^{+/-}$ males (upper panels) and $AR^{+/-}$ females (lower panels) exhibit all the secondary sexual characteristics typical of $AR^{+/+}$ roosters or hens, including comb, body size and tail feathers (**A1-A2, B1-B2;** see Fig. 1). The testicles showed a male-typical size (**A3**) and normal histology of seminiferous tubules (ST) (**A4**), containing mature sperm, indicating a normal reproductive development. Likewise, $AR^{+/-}$ female ovaries contained follicles at various stages of maturity (**B3-B4**), indicating normal ovarian development, which was further supported by their ability to lay eggs regularly (see Fig. 2F). These findings demonstrate that having one functional copy of the AR gene is sufficient to develop sexual phenotypes associated with their respective sexes. Sections of testicles and ovaries were stained with Haematoxylin for three animals per group. Scale bar represents 1 cm for A3, B3 and 200 µm for A4, B4.

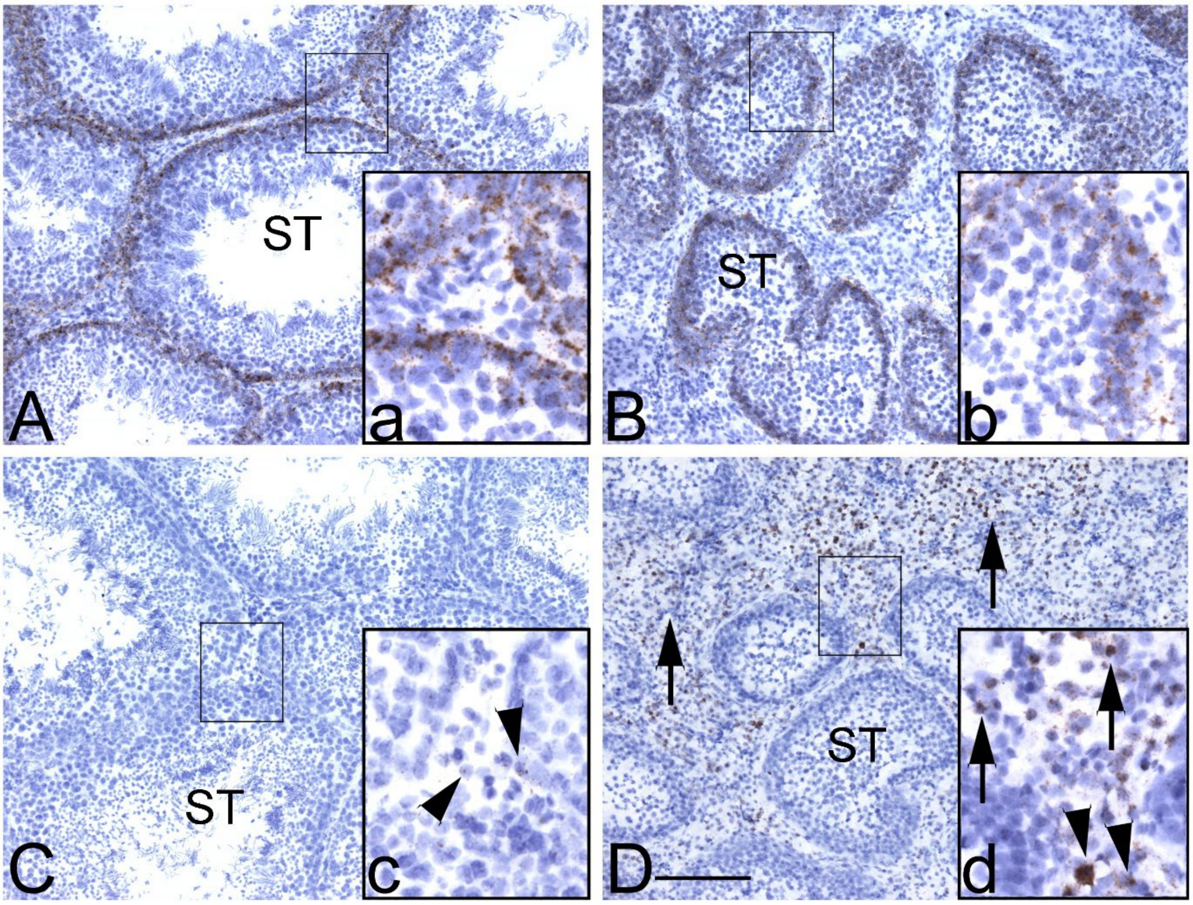


**Fig. S4. Hyperplasia of interstitial tissue including Leydig cell-like cells expressing luteinizing hormone receptors (LHR) in the seminiferous tubules (ST) of homozygous AR$^{-/-}$ males.** Histological analyses, along with the examination of expression of DMRT1 mRNA expression (brownish stain), a marker for Sertoli cells, reveal comparable abundance of Sertoli cells between wild-type (AR$^{+/+}$; **A**) and AR$^{-/-}$ (**B**) males. In contrast, the expression level of LHR mRNA, indicative of Leydig cells, markedly differs between the two genotypes, with low levels in AR$^{+/+}$ (brownish dots indicated by arrowheads in the insert **c** of **C**) and higher in AR$^{-/-}$ (arrowheads in **d** of **D**). The mean densities of dots representing LHR mRNA were 1.63, 1.55 and 1.32 for three AR$^{+/+}$ males and were 4.79, 4.54 and 4.03 for three AR$^{-/-}$ males, respectively. Further, interstitial tissue is massively expended in AR$^{-/-}$ (B, D), best visible in the upper part of D (arrows) and in d (arrows), and contains many LHR mRNA expression cells. In A to D, the small squares indicate the enlarged areas of the inserts a-d. The detection of LHR mRNA and DMRT1 mRNA was performed in three animals per group using the RNAscope method and counterstained with Hematoxylin (bluish). The scale bar represents 150 µm for A-D and 50 µm for a-d.

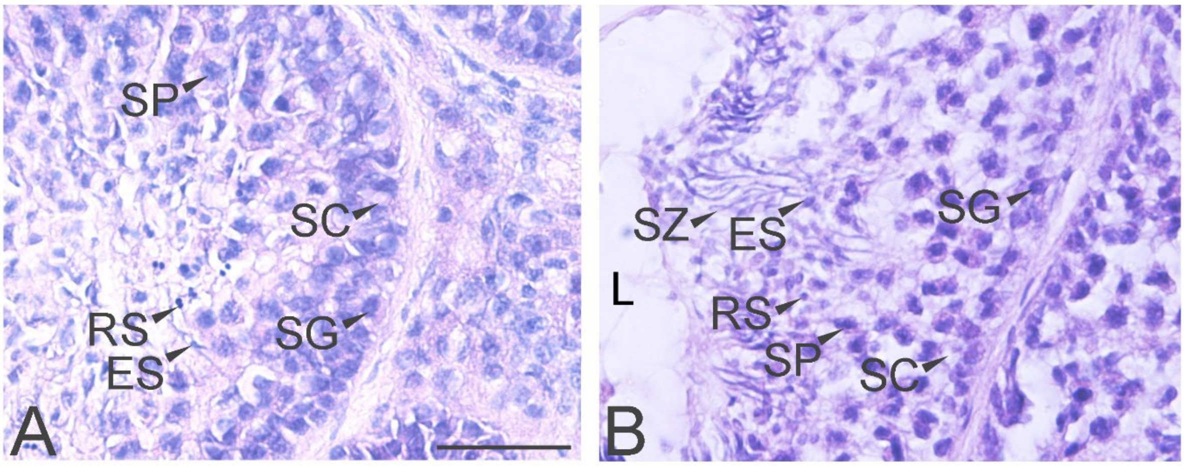


**Fig. S5**. **Comparison of seminiferous tubules in AR$^{-/-}$ and AR$^{+/+}$ testicles.** The seminiferous tubules of the AR$^{-/-}$ testicles (n = 3; **A**) lack a lumen and spermatogenesis is interrupted compared to the AR$^{+/+}$ testicles (n = 3; **B**). In A, only few round spermatids (RS) and elongated spermatids (ES) are observed, with an absence of mature spermatozoa (SZ) typically found in abundance in the wild-type testicle (B). Additional abbreviations: SC = Sertoli cell, SG = spermatogonium, SP = primary spermatocyte. Sections were stained with Hematoxylin. Scale bar represents 80 µm for A and B.

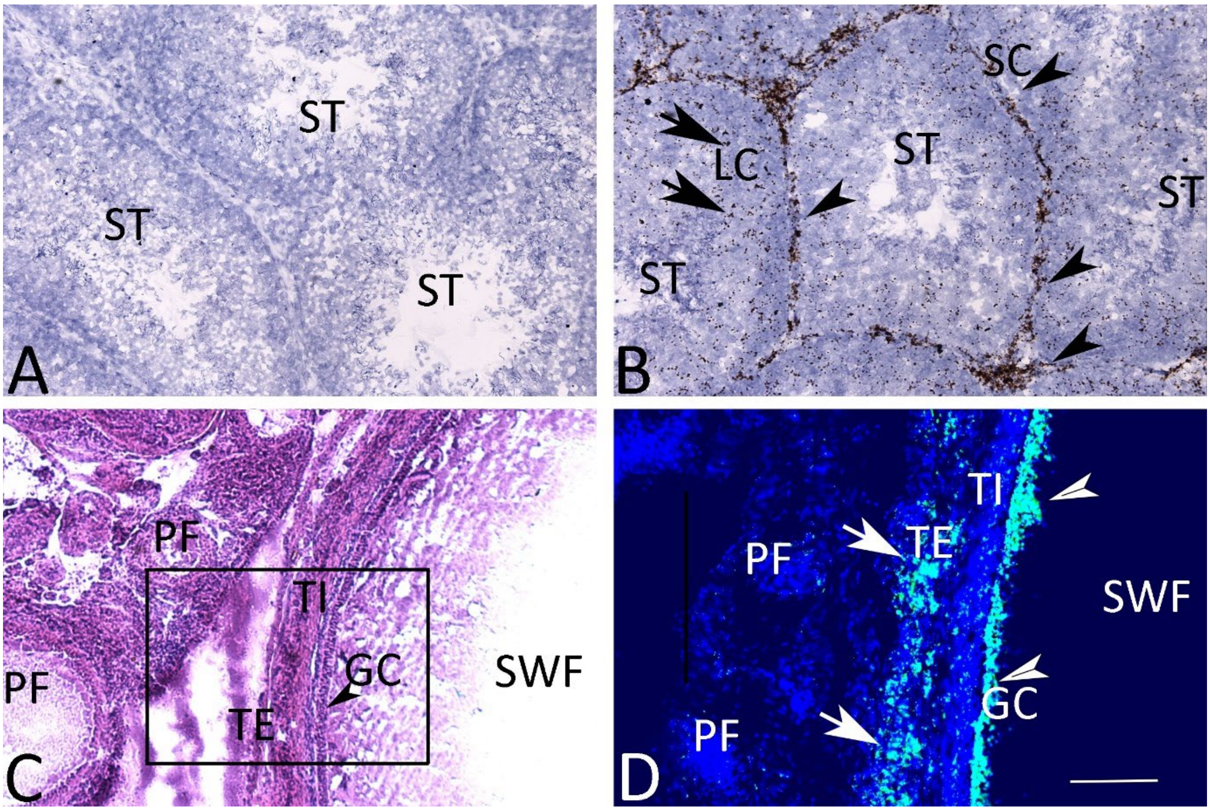


**Fig. S6**. **Localization of androgen receptor (AR) expression in the testicle (upper panels) and ovary (lower panels) of adult chickens.** Hematoxylin staining revealed the presence of seminiferous tubules (ST) in the testicle (**A**) and follicles in the ovary (**C**). In the testicle (**B**), AR mRNA labeled by RNAscope in-situ hybridization (indicated by brown dots) was detected in the Sertoli cells (SC, arrowheads) lining the basal laminae of the seminiferous tubules, as well as in round spermatids and in Leydig cells (LC, arrows). In the ovary (**D**), AR mRNA [appearing as light blue dots, captures using DAPI (dark blue) counterstaining] was strongly expressed in the layer of granulosa cells surrounding the small white follicles (SWF) and small yellow follicles (not shown), along with interstitial cells of the theca externa (TE). Little or no AR mRNA was expressed in primary follicles (PF) and in the theca interna (TI). (D) corresponds roughly to the rectangle shown in (C). For each analysis, three individuals were analyzed. Scale bar represents 100 μm for A to C, and 50 μm for D.

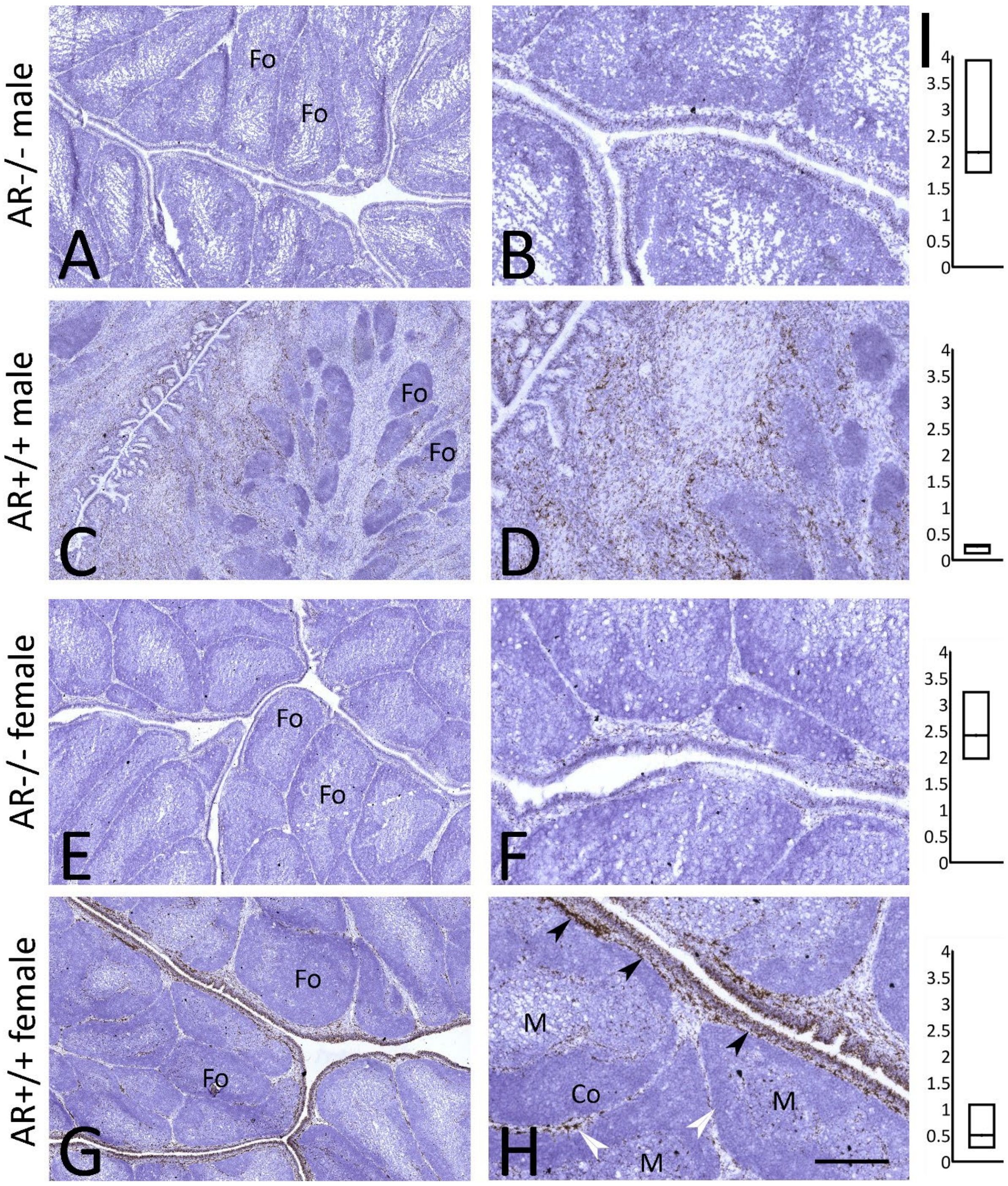


**Fig. S7**. **The normal regression process (involution) of the bursa of Fabricius, which begins during puberty in wild-type** (AR$^{+/+}$) **chickens, is either absent or significantly delayed in homozygous (AR$^{-/-}$) chickens, regardless of sex, at adulthood (20 weeks of age)**. Representative photomicrographs showing the morphology of the bursae of AR$^{-/-}$ (A, B, E, F) and wild-type AR$^{+/+}$ (C, D, G, H) male and female chickens at 20 weeks of age is depicted (n = 3 for each group). In AR$^{+/+}$ males (**C, D**), the bursa shows normal signs of regression, evident through the reduced follicle (Fo) size, the expanded connective tissue and the overall reduced

size (**I**, bursa weight in gram, n = 3 for each group with minimum, median and maximum depicted as box blots, see Tab. S5 for data). Similarly, in $AR^{+/+}$ females, the bursa showed an advanced regressed state in terms of weight, although it still contained plicae with follicles typical of juvenile size (**G, H** and right column; see above for explanation). In contrast, both $AR^{-/-}$ males (**A, B**) and $AR^{-/-}$ females (**E, F**) exhibited limited evidence of bursa regression, both in terms of morphology and overall weight (I; see Fig. 3F), suggesting disrupted development and morphology. The density of follicles appeared higher in $AR^{-/-}$ individuals compared to $AR^{+/+}$ chickens, with $AR^{+/+}$ males (C, D) demonstrating this difference more prominently. We further depict the distribution of AR mRNA labeled cells, visualized as brown dots, in the bursa of the $AR^{+/+}$ males (**D**) and females (**H**). AR mRNA was mainly expressed in the mesenchymal cells of the epithelium of the plicae (black arrowheads in H), in the laminae surrounding the follicles (white arrowheads in H), and in the medulla (M) but not in the cortex (Co) of the follicles. AR-expressing cells are the probable starting point of testosterone-triggered regression of the bursa during ontogeny. Shown are bursa sections stained with Haematoxylin (A-H;), stained for AR mRNA expression in the bursa (C, D, G, H). Scale bar represents 250 µm for A, C, E, G and 100 µm for B, D, F, H.

## Supplementary Tables

**Tab. S1. Transmission rate of chimeras**. The values denote the frequency at which chimeric traits are successfully inherited by off-springs.

| PGC LSL 2-6 Line | Chimera | Total hatched progeny | $AR^{+/-}$ | Male | Female |
|---|---|---|---|---|---|
| AR-/- Clone 4 | 1 | 431 | 11 | 3 | 8 |
| AR-/- Clone 4 | 2 | 34 | 0 | 0 | 0 |

**Tab. S2. The androgen receptor (AR) of the $AR^{-/-}$ chicken is a truncated protein.** Mass spectrometry (LC-MS/MS) analysis of peptides of the AR of the testes of three adult $AR^{-/-}$ and three adult $AR^{+/+}$chicken. The detection of peptides corresponding to exon 1 in all individuals, coupled with the absence of peptides referencing to exons 2 to 8 in the knockouts, indicate the truncation of AR. Therefore, the AR is non-functional in this chicken strain, lacking the DNA-binding domain and the -binding domain.  Listed are all peptides detected in coding exons 1 to 8 of the AR. Numbers indicated the peptide quantities (AU). Empty cells indicate the lack of detection of peptides in a sample.

| | | genotypes | | | | | |
|---|---|---|---|---|---|---|---|
| **Peptide Sequence** | **AR Exons** | **AR+/+** | **AR+/+** | **AR+/+** | **AR-/-** | **AR-/-** | **AR-/-** |
| APRPDAAEPPEPPAPAAFK | 1 | | 273.2 | 260.6 | 326.9 | 355.1 | 283.6 |
| DCFVLPPPAR | 1 | | | 35.7 | | 52.8 | 50.4 |
| EPPPREDCMFALPGGPPR | 1 | 136.4 | 47.0 | 34.3 | | | |
| GSGAEAALAVEVPAGLPLYR | 1 | 55.9 | | 35.3 | | 139.1 | 93.5 |
| VSPPPEEPPGR | 1 | | | | | 272.9 | |
| SELGPWAEGYAGAYGDVR | 1 | 157.2 | 64.8 | 66.4 | | 31.2 | |
| EHILPIDYYFPPQK | 2 | 149.2 | 77.1 | 79.7 | | | |
| TCLICGDEASGCHYGALTCGSCK | 2 | 8.1 | 28.3 | 3.8 | | | |
| CYEAGMTLGAR | 3 & 4 | 247.6 | 140.2 | 89.1 | | | |
| MLYFAPDLVFNEYR | 5 & 6 | 564.3 | 176.8 | 102.7 | | | |
| VLDSVHPIAK | 7 & 8 | 31.5 | 26.3 | 20.8 | | | |
| DLHQFTFDLLIK | 8 | 94.1 | 45.5 | 39.1 | | | |
| AHMVSVDYPEMMAEIISVQVPK | 8 | 368.8 | 95.0 | 112.2 | | | |
| VKPIYFHAE | 8 | 193.6 | 139.5 | 49.1 | | | |

**Tab. S3**. **Plasma levels of testosterone (T) and 17ß-estradiol (E2) during ontogeny of homozygous (AR-/-), heterozygous (AR+/) and wild-type (AR+/+) male (M) and female (F) chickens.** Depending on the day of blood collection, samples were divided into groups ("ages"): 1 week [6-8 days], 3 weeks [15-20 days], 7 weeks [44-52 days], 11 weeks [70-80 days], 15 weeks [100-110 days] and 20 weeks [130-140 days]. Hormone levels are expressed as pg/ml plasma.

| | F | F | F | M | M | M | F | F | F | M | M | M |
|---|---|---|---|---|---|---|---|---|---|---|---|---|
| | AR+/- | AR-/- | AR+/+ | AR+/- | AR-/- | AR+/+ | AR+/- | AR-/- | AR+/+ | AR+/- | AR-/- | AR+/+ |
| Age (weeks) | T | T | T | T | T | T | E2 | E2 | E2 | E2 | E2 | E2 |
| 1 | 15.2 | 15.7 | 13.3 | 27.7 | 19.0 | 64.9 | 15.6 | 15.5 | 12.9 | 15.9 | 22.9 | 15.5 |
| 1 | 40.1 | 14.6 | 13.1 | 22.2 | 19.3 | 11.9 | 16.2 | 16.1 | 15.1 | 15.9 | 19.8 | 10.9 |
| 1 | 24.7 | 26.8 | 12.8 | 24.0 | 19.9 | 12.7 | 27.5 | 16.0 | 7.9 | 15.6 | 20.3 | 8.1 |
| 1 | 22.9 | 14.4 | 15.0 | 30.7 | 15.0 | 14.5 | 14.6 | 16.5 | 9.3 | 15.2 | 14.1 | 9.8 |
| 1 | 12.1 | 56.9 | 14.2 | 21.5 | 16.0 | 17.0 | 8.4 | 16.8 | 7.6 | 15.6 | 12.9 | 9.0 |
| 1 | 13.2 | 14.3 | 13.5 | 62.4 | 27.0 | | 10.0 | 8.8 | 8.0 | 8.2 | 15.0 | |
| 1 | 17.7 | 12.7 | 13.8 | 25.1 | 33.6 | | 11.4 | 8.2 | 7.9 | 9.8 | 15.7 | |
| 1 | 14.8 | 28.0 | 14.2 | 17.2 | 42.0 | | 12.7 | 16.4 | 9.6 | 10.6 | 14.8 | |
| 1 | 15.2 | 16.9 | | 21.9 | 29.2 | | 9.8 | 8.0 | 7.7 | 10.3 | 16.4 | |
| 1 | 15.3 | 15.0 | | 13.6 | 97.0 | | 8.2 | 10.2 | | 10.0 | 14.1 | |
| 1 | 14.1 | 13.6 | | 119.2 | 13.5 | | 8.1 | 12.2 | | 8.1 | 8.8 | |
| 1 | 15.4 | 13.3 | | 29.6 | 16.1 | | 8.6 | 9.7 | | 8.4 | 9.2 | |
| 1 | 14.0 | 12.5 | | 12.0 | 27.2 | | 8.6 | 8.6 | | 10.3 | 9.5 | |
| 1 | 12.0 | | | 34.4 | 23.1 | | | | | 8.6 | 10.5 | |
| 1 | | | | | 15.1 | | | | | 8.1 | 10.8 | |
| 1 | | | | | 13.2 | | | | | 8.4 | 8.2 | |
| 1 | | | | | 14.3 | | | | | 10.1 | 8.3 | |
| 1 | | | | | 13.5 | | | | | 8.2 | 8.1 | |
| 1 | | | | | | | | | | 7.7 | | |
| 3 | 19.5 | 15.7 | 15.8 | 180.0 | 44.3 | 13.6 | 12.3 | 30.4 | 30.7 | 15.5 | 13.3 | 8.5 |
| 3 | 15.0 | 15.5 | 13.5 | 23.5 | 41.8 | 19.0 | 11.7 | 21.1 | 8.2 | 16.6 | 16.1 | 8.1 |
| 3 | 17.9 | 34.5 | 13.1 | 29.4 | 14.4 | 31.3 | 22.3 | 15.5 | 10.1 | 16.2 | 17.1 | 7.6 |
| 3 | 14.1 | 13.4 | 12.1 | 30.9 | 14.3 | | 17.1 | 15.9 | 8.1 | 14.8 | 16.0 | |
| 3 | 13.7 | 12.9 | 13.9 | 118.4 | 59.8 | | 15.9 | 7.5 | 11.0 | 16.7 | 15.0 | |
| 3 | 12.7 | 16.3 | 13.4 | | 82.4 | | 12.5 | 19.4 | 17.0 | 14.1 | 8.4 | |
| 3 | 17.3 | | | | 32.4 | | 12.0 | 10.1 | | 18.2 | 8.0 | |
| 3 | 15.6 | | | | 84.5 | | 11.7 | 9.8 | | 15.6 | 9.4 | |
| 3 | 18.0 | | | | 12.0 | | 9.3 | 13.9 | | 8.9 | 9.7 | |
| 3 | 13.6 | | | | | | 14.8 | 128.9 | | 8.4 | 8.7 | |
| 3 | | | | | | | | | | 9.4 | | |
| 3 | | | | | | | | | | 7.6 | | |
| 7 | 14.6 | 13.1 | 17.0 | 186.5 | 680.6 | 529.5 | 22.8 | 8.6 | 21.3 | 13.4 | 8.2 | 8.2 |

| | | | | | | | | | | | | |
|---|---|---|---|---|---|---|---|---|---|---|---|---|
| 7 | 14.3 | 12.9 | 16.7 | 105.5 | 746.4 | 586.2 | 20.1 | 8.8 | 24.8 | 13.1 | 11.2 | 9.0 |
| 7 | 17.0 | 13.8 | 15.7 | 809.4 | 202.9 | 287.3 | 16.1 | 8.2 | 23.6 | 8.6 | 8.5 | 8.8 |
| 7 | 14.7 | 33.1 | 17.6 | 637.0 | 231.1 | | 36.8 | 14.6 | 32.9 | 8.1 | 9.7 | |
| 7 | 15.3 | 16.8 | 13.2 | 344.3 | 81.7 | | 10.0 | 15.4 | 17.8 | 7.9 | 8.3 | |
| 7 | 15.4 | 25.9 | 12.5 | 145.2 | 92.8 | | 21.9 | 16.7 | 12.9 | 8.0 | 7.9 | |
| 7 | 15.7 | 24.0 | 12.8 | | 65.1 | | 20.2 | 13.7 | 41.0 | 8.2 | 13.9 | |
| 7 | 19.1 | | | | 97.9 | | 17.3 | 20.5 | 13.6 | 8.3 | 15.4 | |
| 7 | | | | | | | | 21.6 | 8.8 | 8.2 | 24.5 | |
| 7 | | | | | | | | | | 15.6 | 13.6 | |
| 7 | | | | | | | | | | 20.6 | | |
| 7 | | | | | | | | | | 15.4 | | |
| 11 | 14.2 | 23.3 | 22.7 | 424.3 | 178.1 | 1403.1 | 34.3 | 8.0 | 39.5 | 13.3 | 8.4 | 8.8 |
| 11 | 13.8 | 4526.6 | 32.0 | 301.8 | 32.3 | 1435.8 | 60.7 | 8.0 | 33.7 | 9.9 | 25.3 | 9.6 |
| 11 | 13.0 | 17.8 | 14.0 | 386.6 | 64.1 | 334.5 | 33.4 | 9.8 | 34.8 | 9.6 | 12.8 | 9.8 |
| 11 | 18.1 | | 41.1 | 1214.0 | 137.7 | | 38.8 | 27.7 | 50.7 | 9.4 | 7.6 | |
| 11 | 19.6 | | 15.1 | 807.0 | 1590.8 | | 25.7 | 34.1 | 67.7 | 9.6 | 8.4 | |
| 11 | 14.6 | | 15.9 | | 366.1 | | 33.6 | 23.5 | 47.8 | 7.6 | 7.6 | |
| 11 | 23.7 | | | | | | 31.7 | | 50.2 | 8.7 | | |
| 11 | | | | | | | | | | 21.9 | | |
| 15 | 169.4 | 20.0 | 48.8 | 4989.5 | 394.5 | 3211.1 | 171.8 | 11.7 | 185.8 | 16.0 | 8.6 | 8.1 |
| 15 | 56.2 | 19.4 | 59.2 | 2546.9 | 302.6 | 618.5 | 121.3 | 19.7 | 121.8 | 9.4 | 10.0 | 10.5 |
| 15 | 272.8 | 28.8 | 64.3 | 6763.4 | 1146.8 | 2595.1 | 141.7 | 31.4 | 237.5 | 187.0 | 8.2 | 14.2 |
| 15 | 80.5 | | 31.2 | 2898.5 | 2641.6 | | 70.4 | 15.0 | 290.9 | 11.1 | 9.4 | |
| 15 | 107.5 | | 85.7 | | 874.7 | | | | 115.2 | 9.2 | 8.0 | |
| 15 | | | | | | | | | 239.3 | 8.7 | 8.4 | |
| 20 | 174.4 | 1677.4 | 77.4 | 609.8 | 2358.5 | 3750.6 | 148.0 | 145.7 | 113.9 | 10.4 | 22.7 | 7.7 |
| 20 | 172.7 | 2630.0 | 65.2 | 4027.7 | 365.0 | 5954.0 | 204.9 | 126.2 | 92.7 | 9.2 | 9.4 | 9.6 |
| 20 | 5097.0 | 13.2 | 38.9 | 1391.6 | 259.1 | 86.4 | 9.7 | 21.8 | 97.8 | 9.6 | 7.9 | 89.3 |
| 20 | 247.0 | 2098.6 | 1317.0 | 1802.0 | 1760.7 | 3310.1 | 151.7 | 68.3 | 9.6 | 10.4 | 13.2 | 9.7 |
| 20 | 230.3 | 1572.8 | 23.9 | | 63.7 | 1806.9 | 249.1 | 180.7 | 14.8 | 13.6 | 56.2 | 9.9 |
| 20 | | 31.1 | | | 282.8 | | | 117.0 | | 13.6 | 9.6 | |
| 20 | | | | | 7560.7 | | | 17.4 | | 18.9 | 9.1 | |
| 20 | | | | | 1311.0 | | | | | | | |

**Tab. S4. Statistical comparison of Statistical analysis of testosterone and estradiol levels during development of male and female chickens**. The original hormone data (see Tab. S3) were Log10 transformed. To analyze the transformed data, the fit model procedure was applied to male testosterone (**A**), female testosterone (**B**), male estradiol (**C**) and female estradiol (**D**), with Standard Least Squares and incorporated Restricted Maximum Likelihood Estimation followed by LS Means Tukey HSD post hoc tests for pairwise comparisons. For each comparison, levels not connected with the same letter are significantly different.

**A. Male testosterone**

| Level | | Least Sq Mean |
|---|---|---|
| AR+/+ [20] | A | 3.5528528 |
| AR+/- [15] | A B | 3.5070352 |
| AR+/- [20] | A B | 3.3581277 |
| AR+/+ [15] | A B C | 3.2574229 |
| AR+/+ [11] | A B C D E | 2.9629145 |
| AR-/- [15] | A B C D | 2.8954080 |
| AR-/- [20] | A B C D | 2.8655733 |
| AR+/+ [7] | B C D E | 2.6701043 |
| AR+/- [11] | C D E | 2.6475163 |
| AR+/- [7] | D E | 2.4272559 |
| AR-/- [7] | D E | 2.3020857 |
| AR-/- [11] | E | 2.2134480 |
| AR-/- [3] | F | 1.4745789 |
| AR+/- [3] | F | 1.4371855 |
| AR+/- [1] | F | 1.4098618 |
| AR+/+ [3] | F | 1.3226035 |
| AR-/- [1] | F | 1.3104567 |
| AR+/+ [1] | F | 1.2772013 |

Fixed Effect Tests

| Source | Nparm | DF | DFDen | F Ratio | Prob > F |
|---|---|---|---|---|---|
| Age group | 5 | 5 | 98.06 | 135.1911 | <.0001* |
| Genotype | 2 | 2 | 33.75 | 7.4002 | 0.0022* |
| Genotype*Age group | 10 | 10 | 99.89 | 2.4029 | 0.0133* |

**B. Female testosterone**

| Level | | Least Sq Mean |
|---|---|---|
| AR+/- [20] | A | 2.5977156 |
| AR-/- [20] | A | 2.5741856 |
| AR+/- [15] | A B | 2.0769216 |
| AR+/+ [20] | A B C | 1.9665139 |
| AR+/+ [15] | B C D | 1.7929210 |
| AR-/- [11] | B C D | 1.6107856 |
| AR+/+ [11] | B C D | 1.3857503 |
| AR-/- [15] | B C D | 1.3521899 |
| AR-/- [1] | D | 1.2466330 |
| AR-/- [3] | C D | 1.2223989 |
| AR+/- [1] | D | 1.2205402 |
| AR+/- [11] | C D | 1.2139520 |
| AR-/- [7] | D | 1.2096720 |
| AR+/- [3] | D | 1.2059112 |
| AR+/- [7] | D | 1.1971883 |
| AR+/+ [7] | D | 1.1763883 |
| AR+/+ [3] | D | 1.1423325 |
| AR+/+ [1] | D | 1.1363516 |

Fixed Effect Tests

| Source | Nparm | DF | DFDen | F Ratio | Prob > F |
|---|---|---|---|---|---|
| Age group | 5 | 5 | 109.8 | 33.1183 | <.0001* |
| Genotype | 2 | 2 | 51.66 | 1.3610 | 0.2654 |
| Genotype*Age group | 10 | 10 | 110 | 2.2569 | 0.0194* |

**C. Male estradiol**

| Level | | Least Sq Mean |
|---|---|---|
| AR+/- [15] | A | 1.2875326 |
| AR+/+ [20] | A | 1.1636307 |
| AR-/- [20] | A | 1.1627957 |
| AR+/- [3] | A | 1.1076823 |
| AR-/- [1] | A | 1.1074666 |
| AR+/- [20] | A | 1.0895790 |
| AR-/- [3] | A | 1.0825544 |
| AR-/- [7] | A | 1.0754673 |
| AR+/+ [11] | A | 1.0628898 |
| AR+/- [11] | A | 1.0612151 |
| AR+/- [7] | A | 1.0390836 |
| AR+/+ [15] | A | 1.0390728 |
| AR+/- [1] | A | 1.0305921 |
| AR+/+ [1] | A | 1.0165213 |
| AR-/- [15] | A | 0.9963163 |
| AR-/- [11] | A | 0.9827095 |
| AR+/+ [7] | A | 0.9483904 |
| AR+/+ [3] | A | 0.9169705 |

Fixed Effect Tests

| Source | Nparm | DF | DFDen | F Ratio | Prob > F |
|---|---|---|---|---|---|
| Age group | 5 | 5 | 100.7 | 1.1709 | 0.3288 |
| Genotype | 2 | 2 | 32.33 | 0.9916 | 0.3820 |
| Genotype*Age group | 10 | 10 | 102.8 | 1.2713 | 0.2565 |

**D. Female estradiol**

| Level | | Least Sq Mean |
|---|---|---|
| AR+/+ [15] | A | 2.2727322 |
| AR+/- [15] | A B | 2.0610381 |
| AR+/- [20] | A B C | 2.0098036 |
| AR-/- [20] | A B C | 1.8586825 |
| AR+/+ [11] | B C D | 1.6545836 |
| AR+/+ [20] | B C D E | 1.6327186 |
| AR+/- [11] | C D E F | 1.5535173 |
| AR+/+ [7] | D E F G | 1.2961725 |
| AR+/- [7] | D E F G | 1.2902779 |
| AR-/- [15] | D E F G | 1.2613041 |
| AR-/- [11] | E F G | 1.1914073 |
| AR+/- [3] | F G | 1.1773375 |
| AR-/- [3] | E F G | 1.1373193 |
| AR-/- [7] | G | 1.1290881 |
| AR+/+ [3] | G | 1.0977288 |
| AR-/- [1] | G | 1.0805052 |
| AR+/- [1] | G | 1.0501663 |
| AR+/+ [1] | G | 0.9665862 |

Fixed Effect Tests

| Source | Nparm | DF | DFDen | F Ratio | Prob > F |
|---|---|---|---|---|---|
| Age group | 5 | 5 | 109.3 | 48.9073 | <.0001* |
| Genotype | 2 | 2 | 32.09 | 13.0464 | <.0001* |
| Genotype*Age group | 10 | 10 | 109.6 | 6.1159 | <.0001* |

**Tab. S5. A. Weight of the bursa of Fabricius (g), testicles (g), comb (g), body (kg), bursa-weight to body-weight ratio (g/kg), testicle-weight to body-weight ratio (g/kg), and comb-weight to body-weight ratio for homozygous (AR-/-) and wild-type (AR+/+) male and/or female chickens at adulthood. B. Eggs laid per week of homozygous (AR-/-), heterozygous (AR+/-) and wild-type (AR+/+) female chickens.**

**Tab. S5a**

| sex | geno type | body weight [kg] | bursa weight [g] | bursa/body weight [g/kg] | testicle weight [g] | testicle/body weight [g/kg] | comb weight [g] | comb/ body weight [g/kg] |
|---|---|---|---|---|---|---|---|---|
| F | AR-/- | 1.19 | 3.23 | 2.73 | | | 0.21 | 0.18 |
| F | AR-/- | 0.94 | 2.41 | 2.56 | | | 0.15 | 0.16 |
| F | AR-/- | 1.14 | 2.1 | 1.84 | | | 0.13 | 0.12 |
| F | AR-/- | 1.5 | | | | | 0.15 | 0.10 |
| F | AR+/+ | 1.45 | 0.5 | 0.35 | | | 4.33 | 2.99 |
| F | AR+/+ | 1.44 | 1.08 | 0.75 | | | 5.23 | 3.65 |
| F | AR+/+ | 1.6 | 0.27 | 0.17 | | | 6.12 | 3.84 |
| F | AR+/+ | 1.64 | | | | | 7.75 | 4.74 |
| F | AR+/+ | 1.7 | | | | | 13.87 | 8.16 |
| M | AR-/- | 1.66 | 2.64 | 1.59 | 0.88 | 0.53 | 0.37 | 0.22 |
| M | AR-/- | 1.73 | 2 | 1.16 | 0.88 | 0.51 | 0.37 | 0.21 |
| M | AR-/- | 1.77 | 3.34 | 1.89 | 0.53 | 0.30 | 0.48 | 0.27 |
| M | AR+/+ | 1.48 | 0.29 | 0.2 | 10.7 | 7.24 | 49.75 | 33.61 |
| M | AR+/+ | 1.86 | 0.13 | 0.07 | 15.0 | 8.09 | 44.14 | 23.80 |
| M | AR+/+ | 1.73 | 0.26 | 0.15 | 14.8 | 8.60 | 33.54 | 19.44 |
| M | AR+/+ | 1.89 | | | | | 70.88 | 37.50 |

**Tab.S5b**

| Geno type | eggs/ week | eggs/ week | eggs/ week | eggs/ week | eggs/ week |
|---|---|---|---|---|---|
| AR+/+ | 4 | 5 | 5 | 6 | 4 |
| AR+/+ | 5 | 3 | 4 | 5 | 5 |
| AR+/+ | 5 | 6 | 7 | 4 | 5 |
| AR+/+ | 3 | 3 | 4 | 5 | 4 |
| AR+/- | 6 | 5 | 5 | 4 | 2 |
| AR+/- | 5 | 5 | 6 | 5 | 6 |
| AR+/- | 4 | 4 | 4 | 5 | 7 |
| AR+/- | 5 | 7 | 5 | 6 | 6 |
| AR-/- | 0 | 0 | 0 | 0 | 0 |
| AR-/- | 0 | 0 | 0 | 0 | 0 |
| AR-/- | 0 | 0 | 0 | 0 | 0 |
| AR-/- | 0 | 0 | 0 | 0 | 0 |

**Tab. S6. Weight of the body (g) and bursa of Fabricius (mg) of homozygous (AR-/-), heterozygous (AR+/-) and wild-type (AR+/+) 18 days old embryos treated with testosterone (T) or untreated (C). Further, the bursa to body weight ratio (mg/g) is given.**

| Genotype | Sex | Body weight [g] | Bursa weight [mg] | Bursa / Body [mg/g] | Treat ment |
|---|---|---|---|---|---|
| AR+/+ | F | 21.1 | 12.5 | 59.3 | T |
| AR+/+ | F | 25.8 | 18.5 | 71.7 | T |
| AR+/+ | F | 21.0 | 14.2 | 67.8 | T |
| AR+/+ | F | 20.7 | 19.7 | 95.3 | T |
| AR+/+ | F | 17.4 | 24.3 | 139.9 | T |
| AR+/+ | F | 20.2 | 33 | 163.5 | T |
| AR+/+ | M | 24.5 | 10.3 | 42.1 | T |
| AR+/+ | M | 20.5 | 18.1 | 88.3 | T |
| AR+/+ | M | 20.1 | 11.6 | 57.8 | T |
| AR+/+ | M | 15.7 | 11.8 | 75.2 | T |
| | | | | | |
| AR-/- | F | 19.6 | 19.7 | 100.7 | T |
| AR-/- | F | 19.1 | 23.1 | 121.2 | T |
| AR-/- | F | 21.7 | 22.5 | 103.9 | T |
| AR-/- | F | 23.4 | 30.8 | 131.5 | T |
| AR-/- | F | 19.6 | 22.8 | 116.4 | T |
| AR-/- | M | 23.5 | 25.9 | 110.1 | T |
| AR-/- | M | 22.8 | 28.2 | 123.9 | T |
| AR-/- | M | 15.5 | 16.9 | 109.1 | T |
| | | | | | |
| AR+/- | F | 16.0 | 15.3 | 95.4 | T |
| AR+/- | F | 20.6 | 17.2 | 83.6 | T |
| AR+/- | F | 20.6 | 18.9 | 91.7 | T |
| AR+/- | F | 16.3 | 14.3 | 88.0 | T |
| AR+/- | F | 22.4 | 23.2 | 103.6 | T |
| AR+/- | F | 20.6 | 18.7 | 90.9 | T |
| AR+/- | F | 18.4 | 17.3 | 94.1 | T |
| AR+/- | M | 20.0 | 23.8 | 119.0 | T |
| AR+/- | M | 18.7 | 17.7 | 94.7 | T |
| AR+/- | M | 20.5 | 24.8 | 120.8 | T |
| AR+/- | M | 18.5 | 19.6 | 105.8 | T |
| AR+/- | M | 19.7 | 19.1 | 97.1 | T |
| AR+/- | M | 29.8 | 28.1 | 94.3 | T |

| Genotype | Sex | Body weight [g] | Bursa weigh t [mg] | Bursa / Body [mg/g] | Treat ment |
|---|---|---|---|---|---|
| AR+/+ | F | 22.6 | 55.7 | 247.0 | C |
| AR+/+ | F | 24.4 | 46.8 | 192.2 | C |
| AR+/+ | F | 20.7 | 27 | 130.5 | C |
| AR+/+ | F | 24.2 | 48.6 | 200.6 | C |
| AR+/+ | M | 21.8 | 28 | 128.3 | C |
| AR+/+ | M | 21.1 | 24.8 | 117.4 | C |
| AR+/+ | M | 21.5 | 23.1 | 107.4 | C |
| AR+/+ | M | 20.0 | 41.1 | 205.9 | C |
| AR+/+ | M | 25.4 | 36.5 | 143.8 | C |
| | | | | | |
| AR-/- | F | 26.5 | 43.2 | 163.1 | C |
| AR-/- | F | 20.5 | 28.6 | 139.3 | C |
| AR-/- | F | 22.6 | 29.5 | 130.3 | C |
| AR-/- | F | 22.6 | 12.1 | 53.5 | C |
| AR-/- | F | 21.7 | 38.4 | 177.1 | C |
| AR-/- | F | 20.1 | 32.7 | 162.9 | C |
| AR-/- | F | 18.0 | 28 | 155.4 | C |
| AR-/- | F | 25.8 | 17.2 | 66.6 | C |
| AR-/- | F | 26.0 | 32.1 | 123.4 | C |
| AR-/- | F | 22.6 | 33.1 | 146.5 | C |
| AR-/- | M | 25.1 | 34.7 | 138.2 | C |
| AR-/- | M | 23.7 | 43.9 | 185.0 | C |
| AR-/- | M | 22.6 | 38.4 | 169.6 | C |
| AR-/- | M | 18.7 | 33 | 176.7 | C |
| AR-/- | M | 17.3 | 34.4 | 199.2 | C |
| AR-/- | M | 22.6 | 23.3 | 102.9 | C |
| AR-/- | M | 20.9 | 29.6 | 141.7 | C |
| | | | | | |
| AR+/- | F | 24.2 | 29.5 | 122.1 | C |
| AR+/- | F | 25.5 | 46 | 180.7 | C |
| AR+/- | F | 21.8 | 37.5 | 172.2 | C |
| AR+/- | F | 26.1 | 49.1 | 188.5 | C |
| AR+/- | F | 24.6 | 34.1 | 138.7 | C |
| AR+/- | F | 27.8 | 52.3 | 188.0 | C |
| AR+/- | F | 23.6 | 43.8 | 185.8 | C |
| AR+/- | F | 25.2 | 22.1 | 87.7 | C |
| AR+/- | F | 24.9 | 40.7 | 163.7 | C |
| AR+/- | F | 25.5 | 23.8 | 93.5 | C |

| | | | | | |
|---|---|---|---|---|---|
| AR+/- | F | 20.9 | 24.8 | 118.5 | C |
| AR+/- | F | 13.5 | 15.8 | 116.9 | C |
| AR+/- | F | 24.2 | 28.3 | 117.2 | C |
| AR+/- | F | 18.1 | 22.5 | 124.1 | C |
| AR+/- | F | 20.4 | 30.4 | 148.7 | C |
| AR+/- | F | 22.9 | 53.2 | 232.0 | C |
| AR+/- | F | 24.2 | 48.6 | 200.6 | C |
| AR+/- | F | 22.0 | 38.2 | 173.3 | C |
| AR+/- | F | 20.9 | 36.2 | 172.8 | C |
| AR+/- | F | 24.6 | 41.1 | 167.0 | C |
| AR+/- | M | 24.0 | 50 | 208.2 | C |
| AR+/- | M | 24.4 | 25.1 | 103.1 | C |
| AR+/- | M | 24.1 | 35.4 | 146.8 | C |
| AR+/- | M | 20.4 | 29.4 | 144.1 | C |
| AR+/- | M | 25.3 | 34.7 | 137.1 | C |
| AR+/- | M | 23.2 | 38.9 | 167.9 | C |
| AR+/- | M | 22.9 | 34.3 | 149.9 | C |

**Tab. S7. FSH and LH plasma levels of homozygous knockout (AR-/-) and wild-type (AR+/+) male (M) and female (F) chickens at 20 weeks of age**. Serum follicle stimulating hormone (FSH) and luteinizing hormone (LH) levels were measured using competitive ELISA-based kits. We calculated the mean ± SD for each group. (**A**), in males, the LH level of $AR^{+/+}$ was 0.88 ± 0.60 ng/ml and of $AR^{-/-}$ 1.55 ± 0.99 ng/ml. (**B**), in females, the LH level was 0.57 ± 0.32 ng/ml for $AR^{+/+}$ and 1.67 + 0.81 ng/ml for $AR^{-/-}$. LH levels differed between the $AR^{+/+}$ and $AR^{-/-}$ males and females (two-tailed t-tests: t(24) = 2.086, p = 0.48 for males; t(15) = 3.598, p = 0.013 for females). FSH levels were 0.73 ± 0.29 ng/ml in $AR^{+/+}$ males and 1.30 ± 0.77 ng/ml in $AR^{-/-}$ males, 0.75 ± 0.24 ng/ml in AR+/+ females and 1.74 ± 0.46 ng/ml in $AR^{-/-}$ females. FSH levels differed significantly (two-tailed t-test: males: t(23) = 2.519, p = 0.01; females: t(15) = 5.515, p = 0.0003). The data point marked in grey was removed as an outlier.

**A**

| Sex | Geno type | FSH (pg/ml) | LH (pg/ml) |
|---|---|---|---|
| M | AR+/+ | 1026.3 | 1193.5 |
| M | AR+/+ | 775.7 | 1341.0 |
| M | AR+/+ | 689.3 | 2254.9 |
| M | AR+/+ | 426.7 | 773.6 |
| M | AR+/+ | 351.0 | 212.7 |
| M | AR+/+ | 708.5 | 1126.7 |
| M | AR+/+ | 696.9 | 375.2 |
| M | AR+/+ | 656.6 | 541.6 |
| M | AR+/+ | 1439.5 | 341.9 |
| M | AR+/+ | 593.7 | 223.0 |
| M | AR+/+ | 979.9 | 894.5 |
| M | AR+/+ | 489.0 | 606.3 |
| M | AR+/+ | 702.7 | 1552.7 |
| M | AR-/- | 876.8 | 1258.8 |
| M | AR-/- | 1412.3 | 2206.3 |
| M | AR-/- | 3227.3 | 3774.6 |
| M | AR-/- | 1508.6 | 1285.0 |
| M | AR-/- | 11469.6 | 2625.4 |
| M | AR-/- | 2220.0 | 1715.7 |
| M | AR-/- | 1546.7 | 2690.6 |
| M | AR-/- | 858.5 | 1182.5 |
| M | AR-/- | 983.0 | 660.6 |
| M | AR-/- | 855.6 | 653.6 |
| M | AR-/- | 410.2 | 607.9 |
| M | AR-/- | 879.5 | 570.9 |

**B**

| Sex | Geno type | FSH (pg/ml) | LH (pg/ml) |
|---|---|---|---|
| F | AR+/+ | 383.5 | 692.2 |
| F | AR+/+ | 984.3 | 640.0 |
| F | AR+/+ | 855.1 | 1015.8 |
| F | AR+/+ | 815.1 | 971.3 |
| F | AR+/+ | 989.2 | 256.6 |
| F | AR+/+ | 799.1 | 147.9 |
| F | AR+/+ | 393.3 | 370.5 |
| F | AR+/+ | 760.9 | 438.7 |
| F | AR-/- | 1939.7 | 1728.5 |
| F | AR-/- | 1725.6 | 3447.0 |
| F | AR-/- | 1654.3 | 1185.3 |
| F | AR-/- | 1939.7 | 1129.1 |
| F | AR-/- | 1023.3 | 878.9 |
| F | AR-/- | 2105.3 | 1371.9 |
| F | AR-/- | 970.7 | 1567.6 |
| F | AR-/- | 2215.5 | 2482.0 |
| F | AR-/- | 2058.0 | 1237.8 |
