# Supplementary material for: Unveiling the critical role of androgen receptor signaling in avian sexual development
Source: Nat Commun. 2024 Oct 17;15:8970. doi: 10.1038/s41467-024-52989-w (PMC11487053; doi:10.1038/s41467-024-52989-w)
Supplement: Supplementary file 3 — Reporting Summary [file 41467_2024_52989_MOESM3_ESM.pdf]

nature portfolio

Corresponding author(s): Manfred Gahr

Last updated by author(s): 2024/09/12

Reporting Summary

Nature Portfolio wishes to improve the reproducibility of the work that we publish. This form provides structure for consistency and transparency in reporting. For further information on Nature Portfolio policies, see our Editorial Policies and the Editorial Policy Checklist.

Statistics

For all statistical analyses, confirm that the following items are present in the figure legend, table legend, main text, or Methods section.

n/a	Confirmed	
☐	☒	The exact sample size (*n*) for each experimental group/condition, given as a discrete number and unit of measurement
☐	☒	A statement on whether measurements were taken from distinct samples or whether the same sample was measured repeatedly
☐	☒	The statistical test(s) used AND whether they are one- or two-sided<br>*Only common tests should be described solely by name; describe more complex techniques in the Methods section.*
☐	☒	A description of all covariates tested
☐	☒	A description of any assumptions or corrections, such as tests of normality and adjustment for multiple comparisons
☐	☒	A full description of the statistical parameters including central tendency (e.g. means) or other basic estimates (e.g. regression coefficient) AND variation (e.g. standard deviation) or associated estimates of uncertainty (e.g. confidence intervals)
☐	☒	For null hypothesis testing, the test statistic (e.g. *F*, *t*, *r*) with confidence intervals, effect sizes, degrees of freedom and *P* value noted<br>*Give P values as exact values whenever suitable.*
☒	☐	For Bayesian analysis, information on the choice of priors and Markov chain Monte Carlo settings
☐	☒	For hierarchical and complex designs, identification of the appropriate level for tests and full reporting of outcomes
☒	☐	Estimates of effect sizes (e.g. Cohen's *d*, Pearson's *r*), indicating how they were calculated

Our web collection on statistics for biologists contains articles on many of the points above.

Software and code

Policy information about availability of computer code

Data collection	LC-MS/MS data acquisition: Raw data were processed using the Spectronaut 17.0 in directDIA+ (library-free) mode.
Data analysis	LC-MS/MS data analysis: The match-between-run option was enabled, and peptides/proteins were quantified across samples using the label-free quantification (MaxLFQ) at the MS2 level.<br>FSH and LH plasma levels: The hormone concentrations in ng/ml were then calculated using the standard curve generated by a four-parameter logistic curve (4-PL) fitted with GainData® software.<br>Histology and RNAscope in-situ hybridization: For measurement of the diameter of the seminiferous tubules and the quantification of LHR mRNA we used ImageJ.<br>Sound analysis: Four-hour periods of sound recordings per animal and time point were analyzed with the software Audacity.

For manuscripts utilizing custom algorithms or software that are central to the research but not yet described in published literature, software must be made available to editors and reviewers. We strongly encourage code deposition in a community repository (e.g. GitHub). See the Nature Portfolio guidelines for submitting code & software for further information.

Data

Policy information about availability of data

All manuscripts must include a data availability statement. This statement should provide the following information, where applicable:

- Accession codes, unique identifiers, or web links for publicly available datasets
- A description of any restrictions on data availability
- For clinical datasets or third party data, please ensure that the statement adheres to our policy

Source data used in graphs and in the text are available in tables S1 to S7 of the Supplementary Information and tables S2 to S7 in Excel Format in the Source Data file. Table S1 shows transmission rate of the knockout in the chimeras. Table S2 shows the Mass spectrometry (LC-MS/MS) analysis of peptides of the AR of the testes of three adult homozygous (AR-/-) and three adult wild-type (AR+/+) chicken. Table S3 shows the plasma levels of testosterone and 17ß-estradiol during ontogeny of homozygous (AR-/-), heterozygous (AR+/) and wild-type (AR+/+) male (M) and female (F) chickens. Table S4 summarizes the of statistical analysis of testosterone and estradiol levels during development of male and female chickens. Table S5A shows the weight of the bursa of Fabricius, of the testicles, of the comb, the body weight, the bursa-weight to body-weight ratio, the testicle-weight to body-weight ratio, and the comb-weight to body-weight ratio for homozygous (AR-/-) and wild-type (AR+/+) male and/or female chickens at adulthood. Table S5B gives the eggs laid per week of homozygous (AR-/-), heterozygous (AR+/-) and wild-type (AR+/+) female chickens. In table S6, the weight of the body and bursa of Fabricius of homozygous (AR-/-), heterozygous (AR+/-) and wild-type (AR+/+) 18 days old embryos treated with testosterone or untreated are listed. Further, the bursa to body weight ratio is given. Table S7 comprises the FSH and LH plasma levels of homozygous knockout (AR-/-) and wild-type (AR+/+) male (Tab. S7A) and female (Tab. S7B) chickens at 20 weeks of age. The AR-/- chicken strain is available upon request.

Research involving human participants, their data, or biological material

Policy information about studies with human participants or human data. See also policy information about sex, gender (identity/presentation), and sexual orientation and race, ethnicity and racism.

Reporting on sex and gender	*Use the terms sex (biological attribute) and gender (shaped by social and cultural circumstances) carefully in order to avoid confusing both terms. Indicate if findings apply to only one sex or gender; describe whether sex and gender were considered in study design; whether sex and/or gender was determined based on self-reporting or assigned and methods used. Provide in the source data disaggregated sex and gender data, where this information has been collected, and if consent has been obtained for sharing of individual-level data; provide overall numbers in this Reporting Summary. Please state if this information has not been collected. Report sex- and gender-based analyses where performed, justify reasons for lack of sex- and gender-based analysis.*
Reporting on race, ethnicity, or other socially relevant groupings	*Please specify the socially constructed or socially relevant categorization variable(s) used in your manuscript and explain why they were used. Please note that such variables should not be used as proxies for other socially constructed/relevant variables (for example, race or ethnicity should not be used as a proxy for socioeconomic status). Provide clear definitions of the relevant terms used, how they were provided (by the participants/respondents, the researchers, or third parties), and the method(s) used to classify people into the different categories (e.g. self-report, census or administrative data, social media data, etc.) Please provide details about how you controlled for confounding variables in your analyses.*
Population characteristics	*Describe the covariate-relevant population characteristics of the human research participants (e.g. age, genotypic information, past and current diagnosis and treatment categories). If you filled out the behavioural & social sciences study design questions and have nothing to add here, write "See above."*
Recruitment	*Describe how participants were recruited. Outline any potential self-selection bias or other biases that may be present and how these are likely to impact results.*
Ethics oversight	*Identify the organization(s) that approved the study protocol.*

Note that full information on the approval of the study protocol must also be provided in the manuscript.

Field-specific reporting

Please select the one below that is the best fit for your research. If you are not sure, read the appropriate sections before making your selection.

☒ Life sciences ☐ Behavioural & social sciences ☐ Ecological, evolutionary & environmental sciences

For a reference copy of the document with all sections, see nature.com/documents/nr-reporting-summary-flat.pdf

Life sciences study design

All studies must disclose on these points even when the disclosure is negative.

Sample size	We used 3 to 10 animals per group, depending on the availability of knockout animals of the various age groups
Data exclusions	In Tab. S7, one outlier was excluded as indicated in the table

Replication	Replication biological replicates (3 to 10) throughout all groups
Randomization	n.a.
Blinding	n.a.

Reporting for specific materials, systems and methods

We require information from authors about some types of materials, experimental systems and methods used in many studies. Here, indicate whether each material, system or method listed is relevant to your study. If you are not sure if a list item applies to your research, read the appropriate section before selecting a response.

Materials & experimental systems

n/a	Involved in the study
☐	☒ Antibodies
☒	☐ Eukaryotic cell lines
☒	☐ Palaeontology and archaeology
☐	☒ Animals and other organisms
☒	☐ Clinical data
☒	☐ Dual use research of concern
☒	☐ Plants

Methods

n/a	Involved in the study
☒	☐ ChIP-seq
☒	☐ Flow cytometry
☒	☐ MRI-based neuroimaging

Antibodies

Antibodies used	Chicken FSH #A74795 and chicken LH #A75578 from Antibodies Com
Validation	Antibodies were validated by Antibodies Com

Animals and other research organisms

Policy information about studies involving animals; ARRIVE guidelines recommended for reporting animal research, and Sex and Gender in Research

Laboratory animals	Lohmann's Selected Leghorn Classic (LSL) chicken line and the derived Androgen Receptor Knockout strain
Wild animals	n.a.
Reporting on sex	We studied male and female knockout and control chicken throughout all experiments, except for the testosterone-induced induction of crowing. Throughout, we indicated if data are male and or female and described sex-specific effects of the knockout
Field-collected samples	na
Ethics oversight	Government of Upper Bavaria under license number ROB 55.2.2532 Vet-02-17-101

Note that full information on the approval of the study protocol must also be provided in the manuscript.

Plants

Seed stocks	*Report on the source of all seed stocks or other plant material used. If applicable, state the seed stock centre and catalogue number. If plant specimens were collected from the field, describe the collection location, date and sampling procedures.*
Novel plant genotypes	*Describe the methods by which all novel plant genotypes were produced. This includes those generated by transgenic approaches, gene editing, chemical/radiation-based mutagenesis and hybridization. For transgenic lines, describe the transformation method, the number of independent lines analyzed and the generation upon which experiments were performed. For gene-edited lines, describe the editor used, the endogenous sequence targeted for editing, the targeting guide RNA sequence (if applicable) and how the editor was applied.*
Authentication	*Describe any authentication procedures for each seed stock used or novel genotype generated. Describe any experiments used to assess the effect of a mutation and, where applicable, how potential secondary effects (e.g. second site T-DNA insertions, mosiacism, off-target gene editing) were examined.*
